# Permian ancestors of Hymenoptera and Raphidioptera

**DOI:** 10.3897/zookeys.358.6289

**Published:** 2013-12-04

**Authors:** Dmitry E. Shcherbakov

**Affiliations:** 1 Borissiak Paleontological Institute, Russian Academy of Sciences, Moscow 117997, Russia

**Keywords:** Holometabola, Neuropteroidea, Hymenoptera, Raphidioptera, Permian, miniaturization

## Abstract

The origin of Hymenoptera remains controversial. Currently accepted hypotheses consider Hymenoptera as the first side branch of Holometabola or sister-group to Mecopteroidea. In contrast, fossils confirm the idea of Martynov that Hymenoptera are related to Megaloptera and Raphidioptera. Hymenoptera have descended along with Raphidioptera from the earliest Megaloptera, the Permian Parasialidae. A related new family, minute Nanosialidae from the Permian of Russia is supposedly ancestral to Raphidioptera. The fusion of the third ovipositor valvulae is shown to be not a synapomorphy of Neuropteroidea. Parasialids and nanosialids bridge the gap between megalopterans and snakeflies; all can be classified into a single order, Panmegaloptera **nom. n.**, including a new suborder Siarapha for Nanosialidae. The earliest megalopterans and their descendants, Raphidioptera and Hymenoptera, have passed through a “miniaturization bottleneck”, likely a common macroevolutionary mechanism.

## Introduction

The earliest and most primitive Hymenoptera are sawflies (Symphyta) from the Triassic subfamily Archexyelinae of the extant family Xyelidae (Rasnitsyn 1969). Several hypotheses have been proposed regarding their origin: (A) Hymenoptera have descended from an extinct non-holometabolan group—Protoblattoidea (Handlirsch 1906–1908) or Protohymenoptera (now Megasecoptera; Tillyard 1924)—and acquired complete metamorphosis in parallel to other holometabolan groups; such views are now abandoned. (B) Hymenoptera constitute the first side branch of Holometabola, as retaining the unmodified ovipositor (Ross 1965), the view supported by molecular evidence (Wiegmann et al. 2009, Beutel et al. 2011, Trautwein et al. 2012); hymenopterans are derived from the extinct order Miomoptera (Carboniferous–Jurassic), interpreted as the most basal holometabolan group (Rasnitsyn 2002), but no intermediate fossil forms have been found (Miomoptera are supposedly polyphyletic—Shcherbakov 2006, Nel et al. 2012). (C) Hymenoptera constitute a sister-group to Mecopteroidea (Hennig 1969), but the proposed synapomorphies (Kristensen 1975, 1999, Königsmann 1976, Beutel and Vilhelmsen 2007) are inconclusive: eruciform larvae (exceptions: Nannochoristidae and some Trichoptera) with a single pretarsal claw (shared with some Coleoptera; exception: Argidae—Rasnitsyn 1969) and silk produced by the labial glands (shared with Psocoptera); a fully sclerotized floor of the sucking pump in adults (shared with Paraneoptera). (D) Hymenopterans have descended from archaic neuropteroids and show many similarities with Megaloptera and Raphidioptera (Crampton 1924, Martynov 1930, 1937, Ross 1936, 1955).

This latter hypothesis was formulated as follows: “…Hymenoptera evolved from ancestors, somewhat intermediate between Megaloptera, Raphidioptera and Mecoptera” (Martynov 1930). “Not only the number but also the position of these cross-veins [in the hymenopterous wing] is practically the same as in the *Sialis* wing. This similarity seems too great for a simple coincidence and again suggests a close relationship between the Megaloptera and Hymenoptera” (Ross 1936). “All this resemblance in the venation and structure of wings, as well as in other morphological characters, lead us to the conclusion that the whole order Hymenoptera is allied to the order Raphidioptera, and that the venation in the ancestors of Hymenoptera was similar to that in Raphidioptera, but was somewhat simpler. Raphidioptera represent perhaps a conservative side-branch which evolved early from some ancestors closely allied to those of Hymenoptera” (Martynov 1937). “Coleoptera arose from a raphidian-like ancestor… Hymenoptera may have arisen from the same ancestral form as the Coleoptera” (Ross 1955).

The Permian fossils discussed below partly bridge the gaps between megalopterans, snakeflies, and hymenopterans and confirm the neuropteroid nature of the latter.

Like many authors, especially those tracing taxa transforming through time, I follow traditional phylogenetics rather than cladistics and accept both ancestral and terminal taxa (in cladistics, paraphyletic and holophyletic)—these are just two stages in the taxon history, all paraphyletic taxa have once been holophyletic, and *vice versa*, the now holophyletic taxa may eventually turn paraphyletic. As Cavalier-Smith (2010) points out, “Hennigian cladistics emphasizes only lineage splitting, ignoring most other major phylogenetic processes… It has been conceptually confusing and harmed taxonomy, especially in mistakenly opposing ancestral (paraphyletic) taxa” (see also Sharov 1971, Mayr and Bock 2002, Rasnitsyn 2006, Hoerandl and Stuessy 2010, and references therein).

## Materials and methods

The material on the new taxa described herein is deposited at the Borissiak Paleontological Institute, Russian Academy of Sciences (PIN). The fossils were photographed using a Leica MZ9.5 stereomicroscope and Leica DFC420 camera, and imaged without coating with secondary electron (SE) and backscattered electron (BSE) detectors of a Tescan Vega XMU scanning electron microscope. Images were adjusted with Adobe Photoshop CS3. Line drawings were prepared with Inkscape 0.48.

## Results

**Figures 1–4.**
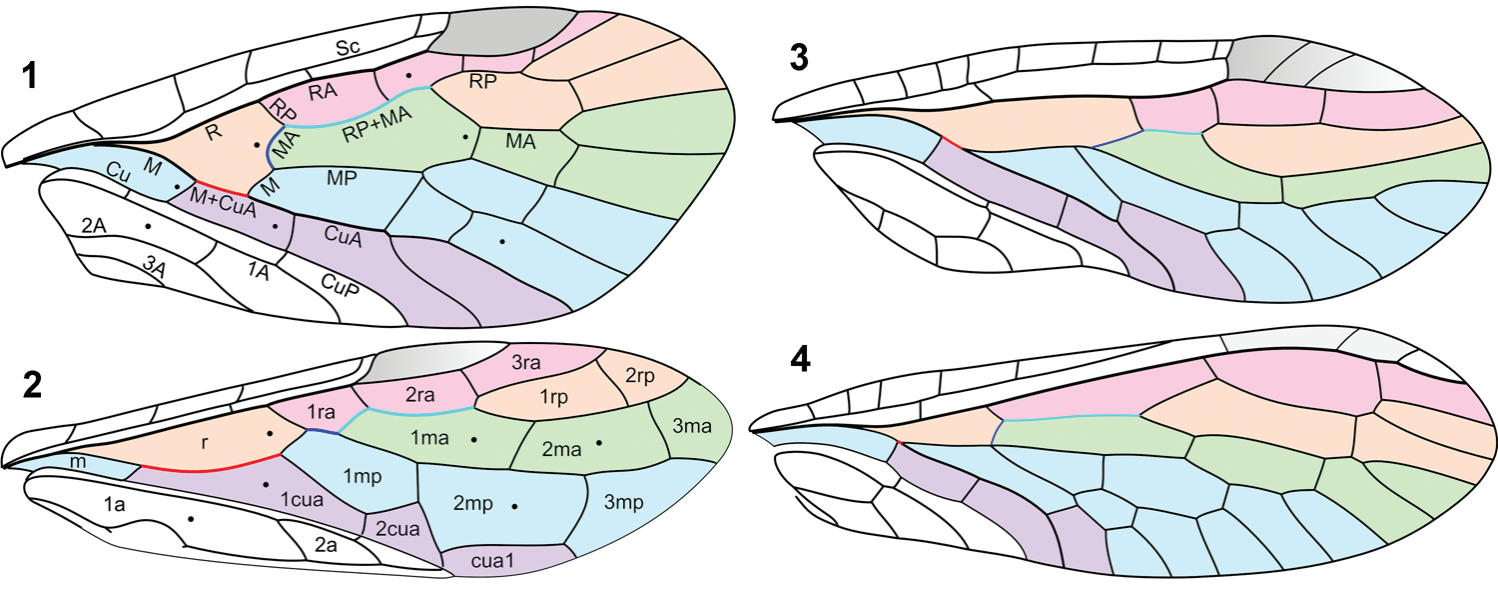
Forewing venation: **1** *Parasialis latipennis* (Parasialidae; veins named) **2** Xyelidae (based mainly on Triassic *Asioxyela paurura* and *Madygenius primitivus*; cells named) **3** *Nanosialisponomarenkoi* gen. et sp. n. (Nanosialidae) **4** *Grimaldiraphidia* cf. *parvula* (Mesoraphidiidae). Black dots, nygmata. Not to scale.

**Figures 5–6.**
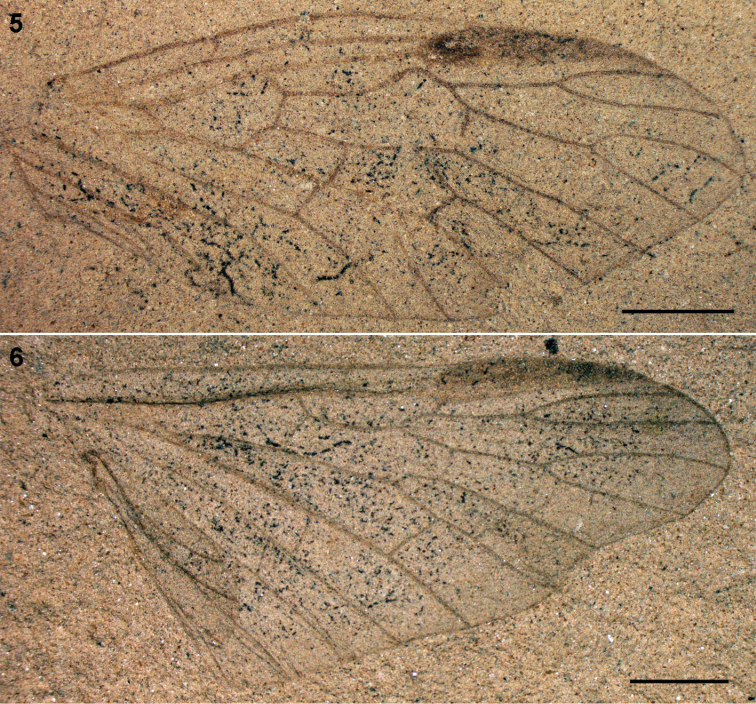
Parasialidae: **5** *Parasialis dissedens*, holotype forewing (mirror image) **6** gen. indet.,hind wing PIN 3353/1073. Scale bars, 2 mm.

The only Megaloptera known from the Palaeozoic are Permian Parasialidae (Ponomarenko 1977), singled out into the suborder Archimegaloptera (Engel 2004). Parasialids, sialids, and symphytans possess stable venation patterns with fixed sets of crossveins and cells (often penta- or hexagonal; Figs 1–3, 5, 6, 8, 11), and their veins and wing membrane are evenly covered with short hairs. Parasialids are also similar to symphytans in a well-developed pterostigma, more distal RP origin (RP base crossvein-like), MP only shortly forked, presence of nygmata (enigmatic, likely glandular, dot-like structures found between veins in various primitive Holometabola and some other pterygotes – Stocks 2008), and also in the long M+CuA anastomosis and RP+MA angled at the base of pterostigma in the forewing. Based on this similarity I suggested that Parasialidae are ancestors of Hymenoptera (Shcherbakov 2006). In contrast to hymenopterans, parasialids retained the pterostigma in their hindwings and therefore were functionally four-winged like all neuropteroids.

**Figures 7–14.**
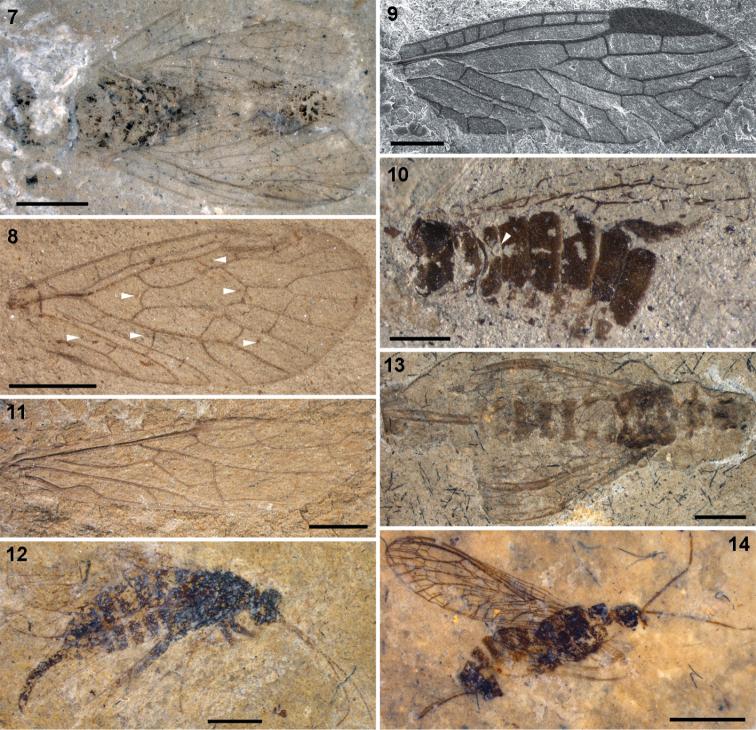
**7–8** Parasialidae: **7** *Parasialis rozhkovi*, holotype male **8** *Parasialis latipennis*, holotype forewing (arrowheads, nygmata) **9–10** Nanosialidae: **9** *Nanosialis ponomarenkoi* gen. et sp. n., holotype forewing PIN 3840/2603A (SEM, SE; mirror image) **10** *Nanosialis ?ponomarenkoi*, body with superimposed wings PIN 3840/2604A (counterpart; arrowhead, incision of 1st abdominal tergite) **11–12** Xyelidae: **11** *Asioxyela paurura* (Archexyelinae) forewing PIN 2785/2491 **12** female Xyelinae indet., PIN 2452/582 **13–14** Mesoraphidiidae: **13** female gen. indet., PIN 2997/2662, halves of ovipositor separated **14** female Nanoraphidiini indet., PIN 2784/1127. Permian of Russia (**7–10**), Triassic of Madygen, Kyrgyzstan (**11**), Jurassic of Karatau, Kazakhstan (**12–14**). Scale bars, 2 mm (**7, 8, 11–14**), 500 µm (**9, 10**).

**Figures 15–22.**
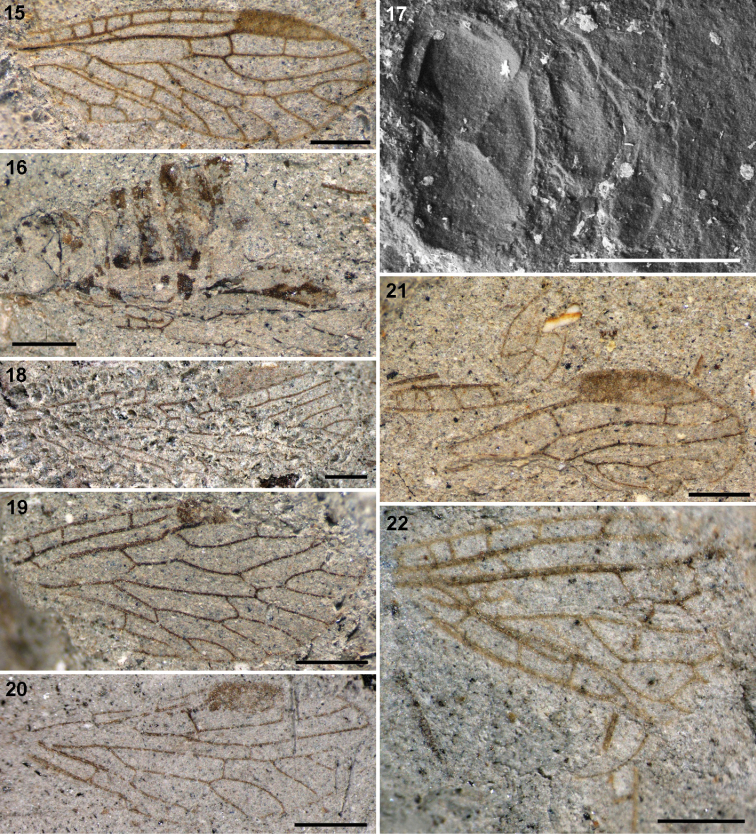
Nanosialidae: **15** *Nanosialis ponomarenkoi* gen. et sp. n., holotype forewing PIN 3840/2603A (mirror image) **16–17** *Nanosialis ?ponomarenkoi* PIN 3840/2604A: **16** body with superimposed wings (part) **17** thorax and base of abdomen (counterpart, SEM, BSE) **18** *Nanosialis bashkuevi* sp. n., holotype hind wing PIN 3840/2633 **19** *Lydasialis micheneri* gen. et sp. n., holotype forewing PIN 3840/2602 **20** *Lydasialis ?micheneri*, hind wing 3840/2601 **21** *Hymega rasnitsyni* gen. et sp. n., holotype forewing PIN 3840/2600 **22** *Raphisialis martynovi* gen. et sp. n., holotype forewing PIN 3840/2009 (mirror image). Scale bars, 500 µm.

The only known body fossil of Parasialidae (Fig. 7) is small, short-bodied, somewhat dorsoventrally depressed, with a large, markedly transverse head, and small, very short pronotum, short legs, and rather short abdomen consisting of well sclerotized segments. Its male genitalia are not unlike those of some megalopterans (and symphytans): without prominent genital capsule, with gonocoxites directed caudad and clavate gonostyles directed mediad (Novokshonov 1993). The overall habitus is rather sawfly-like, except for the broader wings, pterostigma in the hind wing, and homonomous pterothorax.

Differences of Hymenoptera from Megaloptera in the forewing structure are all associated with functional two-wingedness acquired by hymenopterans (Tillyard 1924, Ross 1936): (a) RP+M anastomosis (invariably present in all Triassic Hymenoptera, so the free MA base in some Cenozoic Xyelidae and Siricidae should be a reversal); (b) RP+MA two-branched; (c) MP simple; (d) very long M+CuA anastomosis (free CuA base retained in several families of Symphyta—Rasnitsyn 1969); (e) only two free anal veins (the second represents 2A+3A); (f) two braces between zigzagged CuA and anal veins; (g) RP, MA and MP1 shifted anteriorly, with enlargement of medial cells at the expense of radial cells. These characters correlate with narrowing of the forewing (a–e), strengthening of the forewing anal margin coupled in flight to the hind wing hamuli (f), and costalization of the integrated functional wing (g). The hind wing in Hymenoptera is smaller than the forewing (although the hind wing anal area is well-developed in some Symphyta) and lacks the pterostigma, and the metathorax is smaller than the mesothorax.

The metanotum in Symphyta is equipped with cenchri, which are two blister-like lobes, each interlocking with a field of modified microtrichia (spinarea) on the underside of the forewing anal area in repose (Schrott 1986). This wing-locking mechanism is an elaboration of the microtrichial forewing-metanotum coupling occurring in Neuroptera, Raphidioptera, Sialidae (Riek 1967), some Mecoptera (some Nannochoristidae and Meropeidae; in *Merope* the spinarea is displaced to the upper side of the jugal lobe; Hlavac 1974, Kristensen 1989), and Lepidoptera (Common 1969, Kristensen 2003).

In the very rich Late Permian insect fauna from Isady, northern European Russia (Sukhona River, Vologda Region; Severodvinian, correlated to Wuchiapingian, ~258 million years ago; Bashkuev 2011, Aristov et al. 2013), remarkable minute insects related to parasialids have been discovered, described here as a new family.

### Order Panmegaloptera nom. n.

(=Megaloptera s.l., i.e. *sensu* Latreille, 1802) 

#### Composition.

Four suborders: Archimegaloptera, Megaloptera s.str., Siarapha subordo n., Raphidioptera.

### Suborder Archimegaloptera

Engel, 2004

#### Diagnosis.

As for the family.

#### Composition.

Parasialidae Ponomarenko, 1977

### Family Parasialidae

Ponomarenko, 1977

http://species-id.net/wiki/Parasialidae

#### Diagnosis.

Medium-sized insects (wings 6.5–17 mm long). Sc joining base of pterostigma; RP origin rather distal; RP and MA deeply forked; rp-ma crossvein present; MP once forked (much beyond R fork). Free base of MA developed as crossvein originating from M stem. Anal area at least 1/2 wing length. In forewing, RP+MA angled forwards at base of pterostigma, M and CuA forming long anastomosis (ending nearly level to R fork), M arched forwards distad of anastomosis, free base of MA just beyond M+CuA fork, and CuA forked. In hind wing (Fig. 6), M and CuA connected by very oblique arculus, CuA simple, and anal area variable (narrow in smaller species and broadened in larger species). Nygmata present. Veins and wing membrane evenly covered with short hairs. Short-bodied, somewhat dorsoventrally depressed. Head large, markedly transverse. Pronotum small, very short; pterothorax homonomous; legs short. Abdomen rather short, with short, well sclerotized segments. Male genitalia without prominent genital capsule, gonocoxites directed caudad, clavate gonostyles directed mediad.

#### Composition.

*Parasialis* Ponomarenko, 1977 (Lower to ?Upper Permian of Eurasia; 4 species; Figs 1, 5, 7, 8), *Sojanasialis* Ponomarenko, 1977 (Middle Permian of Soyana; monobasic).

#### Remarks.

In the wing structure Parasialidae are similar to Sialidae, but in the latter the R and MP forks are more proximal in the forewing, and the nygmata are absent.

The hind wings of Parasialidae differ from the forewings in the basal mcu crossvein (arculus) developed instead of M+CuA anastomosis, and CuA unbranched. The hind wing anal area is expanded, with up to six unbranched anal veins in larger parasialids, but relatively small in the smallest parasialid, *Parasialis rozhkovi* (likewise in Sialidae the extent of the hind wing anal area depends on the body size and abdomen mass, so that e.g. in males of smaller species of *Indosialis* the fore and hind wings have anal areas of equal size).

### Suborder Siarapha subordo n.

#### Diagnosis.

As for the family.

#### Composition.

Nanosialidae fam. n.

### Family Nanosialidae fam. n.

http://zoobank.org/1CEA1470-7BCD-44C2-AFCA-E9C55CDADE5B

http://species-id.net/wiki/Nanosialidae

#### Type genus.

*Nanosialis* gen. n.

#### Diagnosis.

Minute insects (wings 2.5–4.5 mm long). Sc joining base of large pterostigma. RP origin distal; ir1 crossvein at base of pterostigma. RP and MA simple (sometimes MA with small fork); rp-ma crossvein absent; MP1 with 3–4, MP2 with 2 branches; CuA apparently simple or with terminal fork. MP fork level to, or just before R fork. M and CuA forming X-junction or very short anastomosis much before R fork (M stem arched towards CuA distad of junction). In forewing, RA sometimes with break at base of pterostigma. Free base of MA developed as crossvein originating from base of MP1 (in hind wing sometimes absent). Hind wing similar to forewing, with narrow anal area. Nygmata absent. Veins beset with strong setae; wing membrane bare. Body short. Pterothorax heteronomous: metanotum smaller and much shorter than mesonotum, without scutoscutellar sutures. Abdomen with short segments; 1st tergite with posteromedian notch.

#### Composition.

Two subfamilies.

#### Remarks.

The body structure is known for the type genus only; the degree of pterothoracic heteronomy and first abdominal tergite division may vary among genera, like with modern genera of some neuropteran families.

In the structure of the proximal wing part (especially in the course of M, oblique direction and position of MA, shape of cells) Nanosialidae are similar to Mesoraphidiidae, but in the latter the pterothorax is always homonomous, anal area is much shorter, pterostigma is displaced distally, and RP+MA is usually more branched.

Among isolated wings of Nanosialidae, those having a shorter anal area, narrower costal area, and more delicate membrane are interpreted as the hind wings.

### Subfamily Nanosialinae subfam. n.

#### Diagnosis.

Pterostigma lanceolate to triangular, moderately elongate, dark. Anal area ~1/2 wing length, with two anal veins.

#### Composition.

*Nanosialis* gen. n., *Lydasialis* gen. n., *Hymega* gen. n.

### Nanosialis gen. n.

http://zoobank.org/50C58857-158A-4870-8C9E-2BF5394D68B0

http://species-id.net/wiki/Nanosialis

#### Type species.

*Nanosialis ponomarenkoi* sp. n.

#### Diagnosis.

Distinct in the long 1mp cell, distal R fork, numerous Sc veinlets, and triangular pterostigma.

#### Composition.

Type species and *Nanosialis bashkuevi* sp. n.

#### Etymology.

Named after Greek *nanos* (dwarf) and *Sialis*; gender feminine.

#### Remarks.

The apparent CuA (probable CuA2) is simple in *Nanosialis ponomarenkoi* forewing, but bears a terminal fork in *Nanosialis bashkuevi* hind wing. This may be an element of the fore/hind wing heteronomy, like in many mesoraphidiids (*Mesoraphidia inaequalis*, *Mesoraphidia pterostigmalis*, etc.).

### Nanosialis ponomarenkoi sp. n.

http://zoobank.org/1E1AC81B-0F3B-45EE-8492-4A94F47DE977

http://species-id.net/wiki/Nanosialis_ponomarenkoi

Figs 3 , 9 , 10 , 15–17 , 23 , 32 , 33

#### Holotype.

Forewing PIN 3840/2603A (part and counterpart).

#### Description.

Forewing 3.0 mm long, elongate (2.7:1), narrowly rounded posterior to MA apex; 7 Sc veinlets; pterostigma distally translucent, with 2 veinlets inside; RP base and crossveins ir1, ir2 transverse, not far separated; ir1 at base of pterostigma; mp cell single, rather long; MP1 3-branched, pectinate backwards; MP2 2-branched; CuA simple; anal area 1/2 wing length; two anal veins (2A+3A with 3 branches), delimiting one anal cell.

Adult PIN 3840/2604A (part and counterpart; head, prothorax, legs, right wings, and apex of abdomen beyond 6th segment, missing; left forewing and hind wing plaited and superimposed, as clearly seen in their pterostigmal areas). Tentatively assigned to the same species on account of similar size and reconstructed forewing venation (differing from the holotype in the larger mp cell and more separated ir1 and ir2 crossveins). Hind wing with well-developed pterostigma. Body as preserved ~2 mm long, somewhat depressed dorsoventrally. Mesoscutum 0.7 mm wide, transverse oval (1.9:1), deeply convex; narrow anterior zone laterally cut off by deep grooves; adjacent third steeply sloped, with semicircular median lobe delimited by arched lines; mesoscutellum low triangular, delimited by deep grooves, with posterior margin slightly arched; mesopostnotum rather narrow. Metascutum 0.55 mm wide, ×1.3 narrower and twice shorter than mesoscutum, subtriangular, more flat, with anterior margin concave, area of metascutellum slightly upturned (no scutoscutellar sutures), posterior margin subangulate; metapostnotum very narrow, arched. Abdomen ~0.7 mm wide, well sclerotized; 1st tergite short (especially medially), broadly sinuate anteriorly, with deep semicircular posterior notch nearly dividing it medially; 2–6th tergites longer, transverse (~2.5:1).

#### Etymology.

Named after the paleoentomologist Alexander Ponomarenko.

**Figures 23–28.**
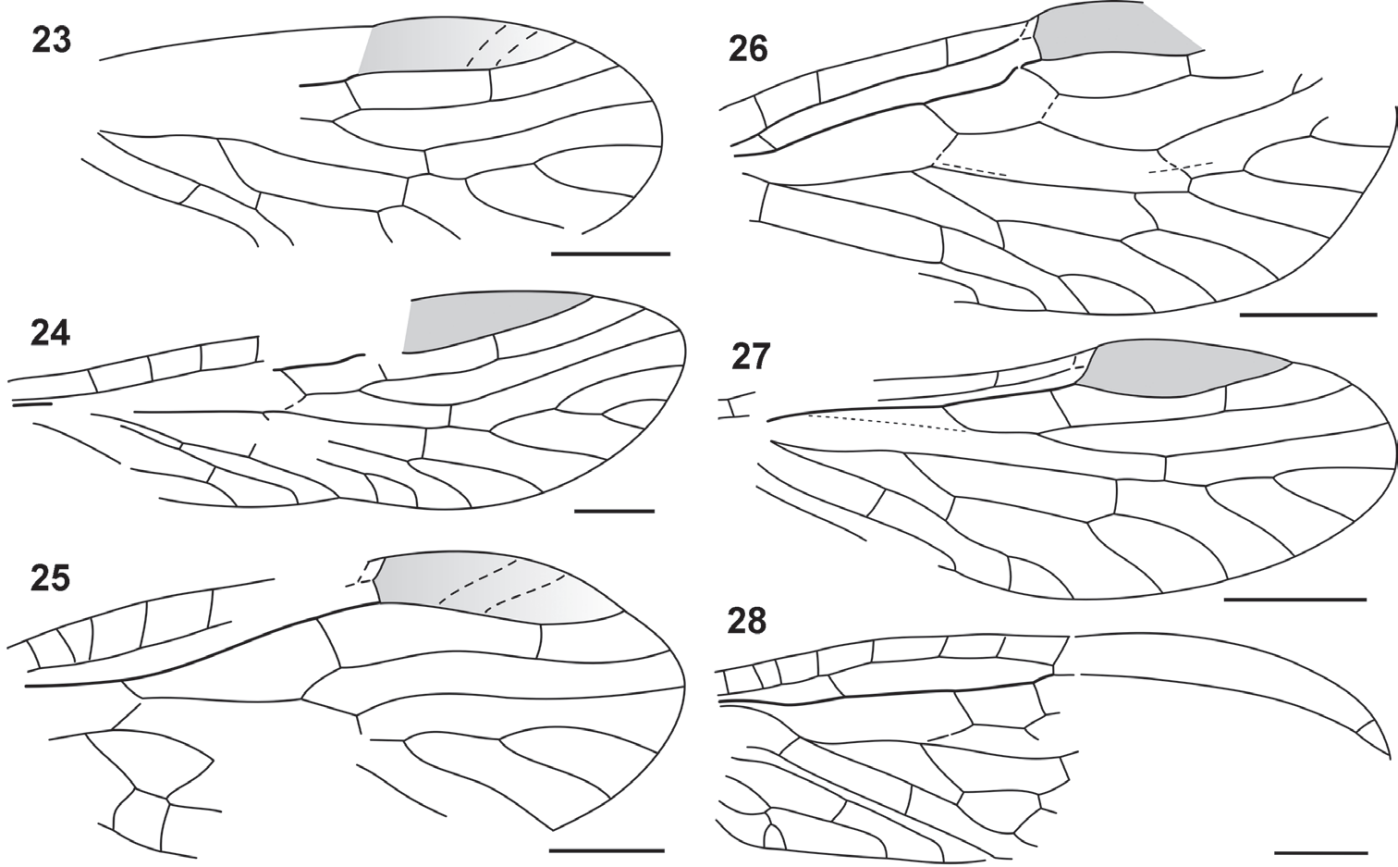
Nanosialidae venation: **23** *Nanosialis ?ponomarenkoi*, forewing, reconstructed **24** *Nanosialis bashkuevi* sp. n., hind wing **25** *Hymega rasnitsyni* gen. et sp. n., forewing PIN 3840/2604A, reconstructed **26** *Lydasialis micheneri* gen. et sp. n., forewing **27** *Lydasialis ?micheneri*, hind wing PIN 3840/2601 **28** *Raphisialis martynovi* gen. et sp. n., forewing, reconstructed. Scale bars, 500 µm.

**Figures 29–34.**
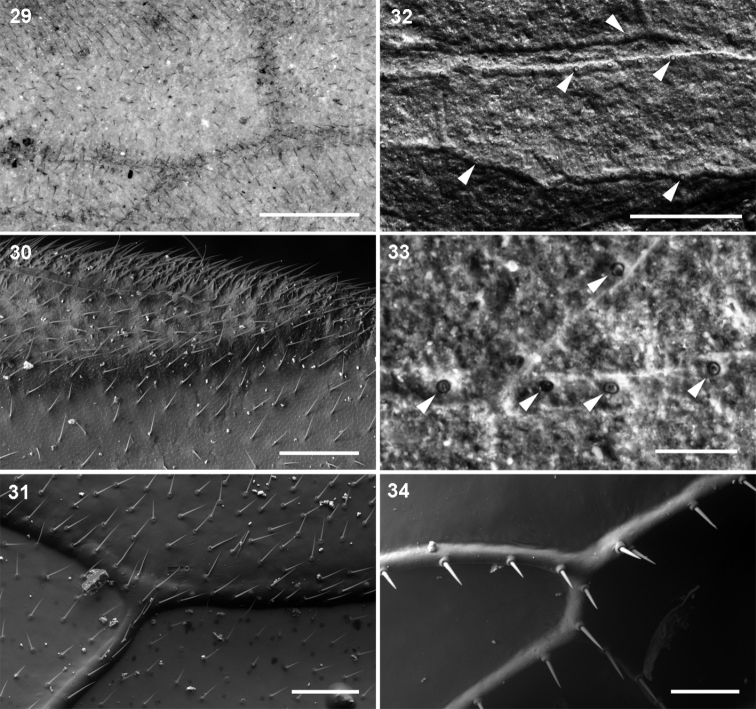
Hairs and setae on forewings: **29–31** uniform hair cover on veins and membrane: **29** Parasialidae, *Parasialis rozhkovi* holotype **30** Sialidae, *Sialis* sp. **31** Xyelidae, *Pleroneura coniferarum* **32–34** strong setae on veins only: **32–33** Nanosialidae, *Nanosialisponomarenkoi* gen. et sp. n., holotype (arrowheads, bases of strong setae on veins) **34** Raphidiidae, *Raphidia* sp., strong erect setae on veins. Recent (**30, 31, 34**); SEM, BSE (**30–34**). Scale bars, 200 µm (**29, 30, 32**), 100 µm (**31, 34**), 50 µm (**33**).

### Nanosialis bashkuevi sp. n.

http://zoobank.org/98E8549D-8C7F-4AF4-A1C9-FEF4892CC044

http://species-id.net/wiki/Nanosialis_bashkuevi

Figs 18 , 24

#### Holotype.

Hind wing PIN 3840/2633 (part and counterpart), interpreted as a hind wing due to the delicate, crimpled wing membrane.

#### Diagnosis.

Distinct from the type species in the larger size and more abundant vein branching.

#### Description.

Hind wing ~4.4 mm long, elongate (~3.2:1), acutely rounded at MA apex; 5? Sc veinlets (four preserved); pterostigma rather evenly suffused, without distinct veinlets; MP1 4-branched; CuA with terminal fork.

#### Etymology.

Named after the paleoentomologist Alexei Bashkuev.

### Lydasialis gen. n.

http://zoobank.org/E9CAA66F-6B78-49F0-B96A-1EEBA27EE643

http://species-id.net/wiki/Lydasialis

#### Type species.

*Lydasialis micheneri* sp. n.

#### Diagnosis.

Very long 1mp cell; few Sc veinlets; in forewing, RA with a break at nodus, and RP section distal to RP+MA desclerotized.

#### Composition.

Monobasic.

#### Etymology.

Named after *Lyda* and *Sialis*; gender feminine.

### Lydasialis micheneri sp. n.

http://zoobank.org/91351955-95EF-402F-8EA2-8A42A49BCFE9

http://species-id.net/wiki/Lydasialis_micheneri

Figs 19 , 20 , 26 , 27

#### Holotype.

Forewing PIN 3840/2602 (the base and most of the anal area missing).

#### Description.

Forewing ~2.8 mm long, broad (~2.4:1), obliquely rounded apically; costal space moderately narrow, with 3 Sc veinlets; pterostigma wide; RA with break at nodus; R fork rather distal; RP base and crossveins ir1, im1, im2, mcu oblique; ir1 at base of pterostigma; RP section between RP+MA and ir1 desclerotized; MA with terminal fork; 1mp cell single, very long; MP1 4-branched, pectinate backwards; MP2 2-branched; CuA with terminal fork beyond mcu.

Hind wing PIN 3840/2601 (base and most of anal area missing, 1A tucked under), interpreted as a hind wing due to the delicate, finely longitudinally wrinkled wing membrane. Tentatively assigned to the same species on account of similar size, few Sc veinlets, a wide pterostigma, and very long 1mp cell. Hind wing ~2.6 mm long, elongate (~2.8:1), narrowly rounded apically; costal space narrower, probably with 3 Sc veinlets; pterostigma wider, rather evenly suffused, without distinct veinlets; no break on RA; only ir1 somewhat oblique; MA simple; MP1 3-branched; MP2 2-branched; CuA with terminal fork.

#### Etymology.

Named after the hymenopterist Charles D. Michener.

### Hymega gen. n.

http://zoobank.org/C7797359-A004-4AFD-9EEF-6D1B071632AE

http://species-id.net/wiki/Hymega

#### Type species.

*Hymega rasnitsyni* sp. n.

#### Diagnosis.

Forewing with R fork and ir1 crossvein proximal, costal space wide, 1mp cell very short (apparently more than one mp cell), and two mcu crossveins.

#### Composition.

Monobasic.

#### Etymology.

Named after Hymenoptera and Megaloptera; gender feminine.

### Hymega rasnitsyni sp. n.

http://zoobank.org/0C81D97C-5273-437E-943A-BCC84D2870B6

http://species-id.net/wiki/Hymega_rasnitsyni

Figs 21 , 25

#### Holotype.

Forewing PIN 3840/2600 (part and counterpart; costal and mp areas torn off and overturned, base and cubitoanal area missing).

#### Description.

Forewing ~3.4 mm long, broad (~2.5:1); costal space wide, with more than 5 Sc veinlets; pterostigma large, distally translucent, with 2 veinlets inside; R fork proximal; RP base short; ir1 oblique, distant from pterostigma; MP1 4-branched; 1mp cell very short; two crossveins mcu.

#### Etymology.

Named after the paleoentomologist Alexander Rasnitsyn.

### Subfamily Raphisialinae subfam. n.

#### Type genus.

*Raphisialis* gen. n.

#### Diagnosis.

Pterostigma sickle-shaped, very elongate. Anal area shorter than 1/2 wing length, with three anal veins.

#### Composition.

Monobasic.

### Raphisialis gen. n.

http://zoobank.org/24742579-4E20-4D18-8BDE-587C52231CDD

http://species-id.net/wiki/Raphisialis

#### Type species.

*Raphisialis martynovi* sp. n.

#### Diagnosis.

Forewing with cell 1mp short (apparently several mp cells) and two mcu crossveins.

#### Composition.

Monobasic.

#### Etymology.

Named after *Raphidia* and *Sialis*; gender feminine.

### Raphisialis martynovi sp. n.

http://zoobank.org/F829E139-1436-4496-8F46-D465BA24BFF3

http://species-id.net/wiki/Raphisialis_martynovi

Figs 22 , 28

#### Holotype.

Forewing PIN 3840/2009 (part and counterpart; anal area and incomplete distal part tucked under).

#### Description.

Forewing ~3.9 mm long, elongate (~3.2:1); costal space moderately narrow, with 7 Sc veinlets; pterostigma long, narrow, sickle-shaped, unpigmented; RA with small fork beyond it; R fork distal; ir1 at base of pterostigma; 1mp cell short (apparently at least three mp cells); two crossveins mcu; anal area shorter than 1/2 wing length; three anal veins (3A with terminal fork), delimiting two anal cells.

#### Etymology.

Named after the paleoentomologist Andrey Martynov.

## Discussion

I consider Nanosialidae derivatives of Parasialidae, not *vice versa*, because of simplified venation, heteronomous pterothorax, the lack of nygmata (so far as known, never restored after being lost), and the M stem arched towards CuA after short M+CuA junction (interpreted as remnants of a longer anastomosis like in parasialids). *Lydasialis* shows a pronounced transverse flexion at the base of the forewing pterostigma, RA having a break (articulation) there (Figs 19, 26), the condition broadly similar to that shared by Parasialidae and Symphyta, which have RP+MA angled there instead. The only known nanosialid body fossil (tentatively assigned to *Nanosialis ponomarenkoi*; Figs 10, 16, 17) resembles parasialids in having a short body and the hind wings retaining the pterostigma,butits metanotum is narrower and much shorter than mesonotum and lacks scutoscutellar sutures, and the first tergite of abdomen is divided medially.

Nanosialids share several characters with hymenopterans: RP+MA two-branched (occasionally MA with short fork, e.g. in aberrant specimens); rp-ma crossvein absent (restored in some Xyelidae: Triassic *Madygenius* and recent *Macroxyela* – Rasnitsyn 1969, Smith and Schiff 1998); very distal RP origin with ir1 crossvein at base of pterostigma; two anal veins; pterothorax heteronomous; 1st abdominal tergite divided. At first glance, Nanosialidae appear even more sawfly-like than Parasialidae, a kind of long-awaited missing link between Megaloptera and Hymenoptera. However, the situation is not so straightforward.

Nanosialidae are distinct from Parasialidae + Hymenoptera and similar to Mesoraphidiidae (Jurassic–Cretaceous; Fig. 4) and other primitive snakeflies in the structure of the proximal wing part (especially in the course of M, position and oblique direction of MA), MP forked proximally (more branched than RP+MA, whereas in hymenopterans MP is simple and RP+MA forked), shape and number of cells, absence of nygmata, and also in the short, stiff, erect setae along veins, and bare wing membrane. Secondary shortening of the M+CuA anastomosis in Nanosialidae and Raphidioptera is associated with shortening of the M stem itself, bringing the MP fork close to MA base; the evidence of a formerly longer anastomosis is the M stem arched close to CuA beyond the M+CuA in Nanosialidae.

The genus *Raphisialis* (Raphisialinae; Figs 22, 28) is additionally similar to mesoraphidiids in the rather short anal area and long, sickle-shaped pterostigma (unpigmented, as in several *Mesoraphidia* spp.). This incompletely known genus is not separated at the family level because the gap between it and *Nanosialis* is partly filled with *Hymega* (Figs 21, 25) having ashort 1mp cell (probably several mp cells) and two mcu crossveins like in *Raphisialis*.

Despite these similarities, Nanosialidae are distinct from Raphidioptera in the longer anal area, more proximal position of pterostigma, less branched RP+MA, and, most importantly, the heteronomous pterothorax, so they cannot be assigned to this order as currently understood. This Permian family is likely to be ancestral to snakeflies, which are still unknown from the Triassic. The striking resemblance between Nanosialidae and Mesoraphidiidae casts doubt on the primitiveness of Jurassic Priscaenigmatidae, considered to be the most basal Raphidioptera (Engel 2002). As evidenced by the pupal tracheation, the CuA1 of Raphidiidae coalesces with MP2 for a distance (Withycombe 1923), so that the apparent CuA is in fact CuA2; this is probably also true of other snakeflies and nanosialids as well.

The aforementioned venation features shared by hymenopterans and nanosialids but not parasialids seem to be associated with miniaturization and likely are homoplasies appearing in closely related lineages, i.e. “underlying synapomorphies” (Saether 1979). Two additional probable homoplasies of Nanosialidae and Hymenoptera are the heteronomous pterothorax (also developed in some functionally four-winged Neuroptera, e.g. Coniopterygidae and Ascalaphidae – Riek 1967) and the first abdominal tergite divided medially (a shallower notch is found also e.g. in Mantispidae – Ferris 1940).

Minute nanosialids, with their veins beset with stiff, erect setae and the wing membrane bare, both like in snakeflies (Figs 32–34), were surely terrestrial. In Parasialidae, the wing membrane and veins are densely covered with short decumbent hairs (Fig. 29). Such a uniform hair cover occurs on the wings of both amphibiotic Megaloptera and terrestrial Hymenoptera (Figs 30, 31) and gives no clue to the life mode of parasialids.

Female genitalia of nanosialids and parasialids are unknown. If Parasialidae were amphibiotic, like the present-day Megaloptera, their ovipositor is likely to have been more or less reduced, suggesting a subsequent restoration of ovipositor in Hymenoptera, Raphidioptera, and possibly in Nanosialidae.

The ovipositor, transformed into a very long, unpaired organ (1st valvulae fused, 3rd valvulae fused dorsally) in living snakeflies, was much more generalized in Mesozoic Mesoraphidiidae, which are sometimes preserved with the left and right halves of the ovipositor separated (in *Siboptera fornicata* (Ren, 1994) and various mesoraphidiids from Karatau, Fig. 13). Therefore, the fusion of the third ovipositor valvulae, previously considered to be a synapomorphy of Neuropteroidea (Mickoleit 1973), was in fact acquired in parallel by some neuropterans and higher snakeflies.

Small Jurassic mesoraphidiids have the ovipositor much shorter than the abdomen, in lateral aspect relatively wide and downcurved (like in some *Xyela* spp. – *Xyela* is from Greek *xyēlē*, curved knife). These small Jurassic snakeflies are short-bodied, with the subquadrate head and short pronotum and abdomen, and look remarkably similar to xyelid sawflies (Figs 12, 14). There are some other notable similarities between snakefies and hymenopterans, including the wasp-like colour pattern in some snakeflies, and the late pupa (in fact, pharate adult) capable of locomotion and with functional mandibles in Xyelidae (Yates and Smith 2009) and Raphidioptera. However, venation differences indicate that the above features have been acquired by snakeflies and sawflies in parallel or inherited from megalopteran ancestors.

**Figure 35.**
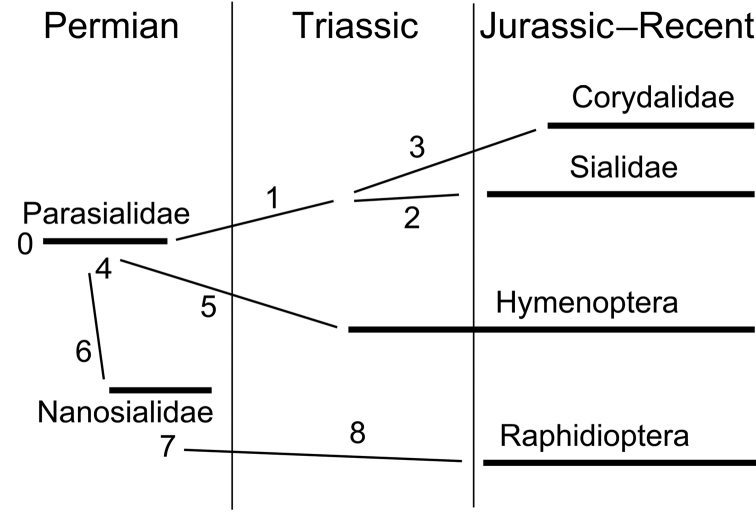
Phylogenetic diagram of Panmegaloptera and Hymenoptera (for characters see Appendix).

Are Parasialidae, the oldest known megalopterans, also the most primitive ones? Megaloptera are still unknown from the Triassic: the only Triassic find ascribed to Megaloptera (Riek 1974) possibly belongs to Polyneoptera (Ansorge 2001). They are rare in the fossil record, likely due to their association with lotic waters, unfavorable for fossil preservation. The two extant megalopteran lineages, Sialoidea and Corydaloidea, are known since the Jurassic (Ansorge 2001, Liu et al. 2012). Sialids and corydalids differ from parasialids in having the vein branching more abundant, which can be interpreted as evidence that the most basal megalopterans are corydalids (because sialids lack nygmata). However, it was suggested (Ponomarenko 1977, 2002) that the early Megaloptera were oligoneurous and that the vein polymerization in Corydalidae is secondary. The discovery of parasialid relatives, oligoneurous Nanosialidae, that are presumably ancestral to the more polyneurous Raphidioptera, further strengthens this hypothesis. Our observations agree with the supposition (Engel and Grimaldi 2008) that Parasialidae may not necessarily have had aquatic larvae and are ancestral to the remaining Megaloptera and Raphidioptera. They furthermore demonstrate that parasialids are ancestral to hymenopterans as well (Fig. 35), placing Hymenoptera among neuropteroid orders.

Parasialidae and Nanosialidae bridge the gap between Megaloptera and Raphidioptera and demonstrate that these two orders can be treated as one. Such was the original concept of Megaloptera (Latreille 1802), but since the currently accepted concept excludes snakeflies, a new name in G. Crampton’s style is proposed here to avoid confusion—Panmegaloptera nom. n. The placement of both amphibiotic insects with a reduced ovipositor and terrestrial insects with a long ovipositor into one order may seem unnatural, but such divergent forms are also found in the closely related order Neuroptera. Nanosialidae, which share several characters with Raphidioptera s.str., are better treated as a new suborder, Siarapha, in Panmegaloptera. Living alderflies, dobsonflies, and snakeflies presumably represent only remnants of the past diversity of archaic neuropteroids with chewing larval mouthparts. It is likely that the extinct panmegalopterans were even more diverse in their life modes, and some of them may have shifted, like sawflies, to palyno- or phytophagy.

The larvae of the most basal hymenopterans supposedly developed in staminate cones of gymnosperms, and their females used their long ovipositors to lay eggs into the cones, like many xyelid sawflies still do (Rasnitsyn 1969). The same life style is reconstructed for the most basal hemipterans, Permian Archescytinidae: their females laid eggs into cones, and nymphs dwelt between the scales (Becker-Migdisova 1985). Indeed, the long, modified ovipositors of some archescytinids have closer analogues among hymenopterans, rather than other hemipterans, suggesting that archescytinids have been ecological predecessors of the earliest hymenopterans (Shcherbakov and Popov 2002). Hymenoptera enter the record in the mid-Triassic, at least 10 Myr after the extinction of Archescytinidae about the Permian–Triassic boundary.

Parasialids and nanosialids are found only in the richest Permian fossil insect sites of Eurasia, being rare in all of them: Chekarda (Lower Permian, Urals)—one *Parasialis rozhkovi* specimen per ~7,000 total insects; Tyulkino (Lower Permian, Urals)—one *Parasialis* specimen per ~550 insects; Soyana (Middle Permian, northern European Russia)—14 Parasialidae specimens (3 species in 2 genera) per ~4,000 insects; Bor Tolgoy (Upper? Permian, southern Mongolia)—two *Parasialis ovata* specimens (Ponomarenko 2000) per ~900 insects, Isady (Upper Permian, northern European Russia)—11 Nanosialidae specimens (5 species in 4 genera) per ~3,500 insects. In the Permian, all of these sites were situated within zones of semiarid or warm temperate climate (Shcherbakov 2008). Why no nanosialids have yet been recorded in other rich Upper Permian entomofaunas? A possible explanation is that these minute terrestrial neuropteroids preyed upon plant lice, like present-day Raphidiidae and various Neuroptera. Indeed, the Isady insect fauna is exceptionally rich with diverse psyllomorphous hemipterans (Aristov et al. 2013).

Miniaturization can be an important source of morphological novelty, in some cases resulting in the origin of higher taxa (Hanken and Wake 1993). The origins of Hymenoptera, of their ancestors Megaloptera, and their close relatives Raphidioptera were likely associated with a “miniaturization bottleneck.” The earliest members of these lineages first underwent reduction in size, leading to incomplete development of many structures (e.g. distal vein branches); later with disappearance of the former size constraints due to changes in the environment or life mode they followed new evolutionary trajectories, regaining some of the lost structures in a highly modified form and evolving new body plans. Naturally, such shifts make tracing the ancestry especially difficult, which can partly explain why the origin of Hymenoptera has long remained a mystery. This mechanism was also responsible for the origin of some other insect orders (e.g. Hemiptera). Still other groups (e.g. Thysanoptera) originated *via* miniaturization but never increased in size again.

After my paper was submitted, an article was published by Nel et al. (2013), likewise stressing the importance of miniaturization in the origin of Hymenoptera and the whole Holometabola plus Paraneoptera. These authors follow the hypothesis B (Hymenoptera are the most basal branch of Holometabola), date the origin of stem hymenopterids at the latest Early Carboniferous (~325 million years ago, Serpukhovian; see their fig. 3) and describe the putative stem hymenopterid *Avioxyela* from the Late Carboniferous (~310 million years ago, Moscovian). The affinities of this fossil, known from fragmentary wings, are highly debatable, it is much more likely to belong to some polyneopteran group (as discussed by Nel et al. 2013 in the supplementary information), and its venation is misinterpreted (the presumed posterior margin of the larger wing is in fact the strengthened costal margin—see their extended data fig. 2). Likewise, other putative Carboniferous paraneopterans and holometabolans described by Nel et al. (2013) may belong elsewhere. For example, *Westphalopsocus*, assigned to Psocodea, is likely to be a nymphal wing pad of a protorthopteran. The data published by Nel et al. (2013) do not affect the conclusions of my paper.

## Supplementary Material

XML Treatment for Panmegaloptera

XML Treatment for Archimegaloptera

XML Treatment for Parasialidae

XML Treatment for Siarapha

XML Treatment for Nanosialidae

XML Treatment for Nanosialinae

XML Treatment for Nanosialis

XML Treatment for Nanosialis ponomarenkoi

XML Treatment for Nanosialis bashkuevi

XML Treatment for Lydasialis

XML Treatment for Lydasialis micheneri

XML Treatment for Hymega

XML Treatment for Hymega rasnitsyni

XML Treatment for Raphisialinae

XML Treatment for Raphisialis

XML Treatment for Raphisialis martynovi

## Appendix

**List of characters:**

**0. Characters of Parasialidae:**

wings homonomous; fore and hind wing not coupled in flight;

pterostigma well developed, oval to lanceolate;

R fork rather distal; RP base crossvein-like;

MP forked distally, with 2 branches;

free MA base crossvein-like, connecting RP and M near bases;

RP+MA twice forked, dichotomous (4-branched);

one or two rp-ma crossveins;

nygmata present;

membrane and veins uniformly covered with short hairs;

**forewing:**

RP+MA angled at base of pterostigma (pronounced nodal flexion);

M+CuA anastomosis moderately long (its apex about level of R fork);

CuA once forked;

Cu base continued with CuP;

anal area reaching ~1/2 wing length;

at least three free anal veins;

**hind wing** anal area enlarged (in larger species) or not;

**pterothorax** homonomous;

presumably also: forewing-metanotum microtrichial coupling.

**1. Synapomorphies of Sialidae + Corydalidae:**

forewing R and MP forks shifted proximad;

ovipositor reduced.

**2. Apomorphy of Sialidae:**

nygmata lost.

**3. Apomorphies of Corydalidae:**

RP+MA pectinate;

**forewing**:

M+CuA anastomosis replaced with crossvein;

Cu base continued with CuA.

**4. Homoplasies (underlying synapomorphies) of Hymenoptera and Nanosialidae + Raphidioptera:**

**forewing:**

R fork shifted distad; crossvein ir1 near base of pterostigma;

RP+MA once forked;

rp-ma crossvein absent;

two free anal veins (2A and 3A fused at least proximally);

**pterothorax** heteronomous;

**1st abdominal tergite** with deep posteromedian notch.

**5. Apomorphies of Hymenoptera:**

wings heteronomous; hind wing without pterostigma, coupled to forewing in flight;

M+CuA anastomosis very long;

**forewing:**

free MA base replaced with RP+M anastomosis;

RP+MA fork shifted distad (to near ir2);

MP simple;

CuA1 shifted posteriad, CuA2 transverse, CuA fork narrow;

two cubitoanal braces between zigzagged CuA and anal veins: crossveins cua-cup and cup-ashifted distad and aligned across reduced distal CuP; CuA2 joining apically fused 1A and 2A+3A;

**pterothorax** markedly heteronomous;

microtrichial areas of metanotum modified into cenchri.

**6. Synapomorphies of Nanosialidae + Raphidioptera:**

pterostigma enlarged;

nygmata absent;

membrane bare; short, stiff, erect setae along veins;

M+CuA anastomosis much shortened or replaced with x-junction;

MP fork shifted proximad, MP with 5–6 branches (more branched than RP+MA);

free MA base connecting RP near base and MP near fork;

CuA1 anastomosing to MP2;

short free 3A sometimes restored (Raphisialinae, Inocelliidae);

**hind wing** anal area always small.

**7. Synapomorphies of Raphisialinae + Raphidioptera:**

pterostigma sickle-shaped, elongate;

anal area somewhat shortened.

**8. Apomorphies of Raphidioptera:**

RP+MA usually twice forked, dichotomous (no less than 4, rarely 3 branches);

at least one rp-ma crossvein;

pterostigma shifted distad;

anal area much shortened;

**pterothorax** always homonomous.
